# Computational model of medial temporal lobe epilepsy

**DOI:** 10.1186/1471-2202-16-S1-P144

**Published:** 2015-12-18

**Authors:** Sora Ahn, Sangbeom Jun, Hyang Woon Lee, Seungjun Lee

**Affiliations:** 1 Department of Electronics Engineering, Ewha Womans University, Seoul, 120-750, Korea; 2 Department of Neurology, Ewha Womans University, Seoul, 120-750, Korea

## 

Temporal lobe epilepsy represents a high proportion of whole epilepsy patients. Medial temporal lobe epilepsy (MTLE) is generated from internal structures like hippocampus, and patients with MTLE are poorly controlled by antiepileptic drugs [1]. Recently, deep brain stimulation (DBS) that is to control seizure activity by stimulating epileptic zone is receiving attention as a new treatment of epilepsy. However, the exact mechanisms are still unclear and the current method is being developed relying on clinical experiences. Consequently, researches for etiology of disease along with seizure suppress mechanisms by electrical stimulation are very significant. These studies would be best progressed with complementary cooperation between in-vitro and in-vivo experiments, and computer simulations using a computational model.

In this paper, we propose a hippocampal network model which portrays seizure-like events (SLEs) recorded in in-vitro experiments. The model is composed of excitatory and inhibitory neurons interconnected following the well-known synaptic pathway to form a small world network [2]. Each neuron is descripted by Izhikevich's model [3] and synaptic current is calculated based on conductance of a receptor. Short-term and long-term plasticity are also applied to every synapse [4]. SLEs induced by 4-AP are divided into three regions according to time-frequency features. The first region is transition to ictal region by excitatory GABAergic drive [5], the second region is tonic firing region by synchronization due to recurrent excitation between principle neurons [6], and the last region is clonic bursting and termination region by GABA-mediated inhibitory mechanisms [1]. Proposed model faithfully reproduces these phenomena by controlling synaptic input gain.

**Figure 1:**
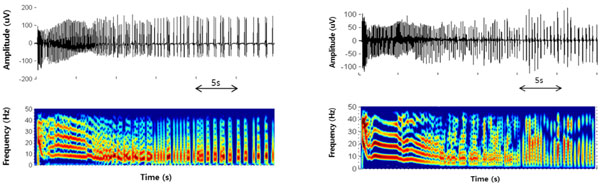
**Recording data (left) and simulation result (right) of seizure-like events in entorhinal cortex**.

The effectiveness of the model is confirmed by comparing the simulation results with experimental data which were recorded in rat hippocampal slice in 4-AP bath application using micro-electrode array (MEA). Below Figure 1 are time domain signals generated from computer model and recorded in in-vitro measurement, respectively.
